# Climate change and alpine-adapted insects: modelling environmental envelopes of a grasshopper radiation

**DOI:** 10.1098/rsos.211596

**Published:** 2022-03-02

**Authors:** Emily M. Koot, Mary Morgan-Richards, Steven A. Trewick

**Affiliations:** Wildlife and Ecology Group, Massey University, Palmerston North, New Zealand

**Keywords:** alpine, climate change, ecological niche modelling, ensemble modelling, biomod2, fragmentation, FRAGSTATS

## Abstract

Mountains create steep environmental gradients that are sensitive barometers of climate change. We calibrated 10 statistical models to formulate ensemble ecological niche models for 12 predominantly alpine, flightless grasshopper species in *Aotearoa* New Zealand, using their current distributions and current conditions. Niche models were then projected for two future global climate scenarios: representative concentration pathway (RCP) 2.6 (1.0°C rise) and RCP8.5 (3.7°C rise). Results were species specific, with two-thirds of our models suggesting a reduction in potential range for nine species by 2070, but surprisingly, for six species, we predict an increase in potential suitable habitat under mild (+1.0°C) or severe global warming (+3.7°C). However, when the limited dispersal ability of these flightless grasshoppers is taken into account, all 12 species studied are predicted to suffer extreme reductions in range, with a quarter likely to go extinct due to a 96–100% reduction in suitable habitat. Habitat loss is associated with habitat fragmentation that is likely to escalate stochastic vulnerability of remaining populations. Here, we present the predicted outcomes for an endemic radiation of alpine taxa as an exemplar of the challenges that alpine species, both in New Zealand and internationally, are subject to by anthropogenic climate change.

## 1. Introduction

Global climate change, whether driven by anthropogenic pollution or Milankovitch cycles, is expressed unevenly across the landscape. In particular, latitude and elevation influence the extent and rate of change in local conditions [1]. Where these gradients intersect in the alpine zone, organisms that endure diurnal and seasonal extremes in temperature and water availability, high winds and elevated UV-radiation during a contracted growth season are exposed to rapid environmental shifts. Among animals, the survival and reproduction of ectotherms is likely to be especially sensitive to displacement of climate isoclines [2,3].

Although most ecosystems will be impacted in some way, arctic, alpine and boreal biomes are predicted to be particularly vulnerable to the ecological impacts of anthropogenic climate change when compared with other systems [4–6]. Growing evidence suggests that global warming results in rapid temperature increases at higher elevations [7]. This is reflected by rising treelines and snowlines, and reduction in snowpack depth and longevity [8–10]. For alpine species, the consequences of such changes include upward shifts in both lower and upper elevational range limits where the landscape permits, increasing inter-species competition at higher elevation, reduction in richness at lower elevation and shifts in phenology [11–14].

Alpine habitat exists at increasing elevations from the poles towards the equator and in total is estimated to currently cover approximately 2.64% of Earth's total land area outside Antarctica (3.56 Mkm^2^) [15,16]. Temperature decreases by approximately 0.65°C for every 100 m of elevation subject to geographic, seasonal and diurnal variance [17,18]. In mountainous areas, this results in the stratification of conditions that is most evident in the abrupt zonation of alpine vegetation, including a clearly defined upper limit to trees on most mountains [19,20]. As a consequence, mountain environments are a sensitive barometer of global climate change, which can yield rapid shifts in the distribution and connectivity of alpine ecosystems [21,22].

The limited and patchy nature of montane topography limits opportunities for alpine adapted organisms to disperse and/or shift their ranges and for alpine environments to persist in the face of global warming [22,23]. For alpine specialists, habitat is typically isolated by an inhospitable low-elevation sea across which dispersal may be limited [24,25]. Fragmentation and patch size reduction, when coupled with poor dispersal ability, will undoubtedly negatively affect the ability of species to track and/or shift their range under future climatic change pressure.

Predicting species responses to global warming, while encompassing all relevant variables is impractical [26,27], but a tool that can be used to explore some facets is ecological niche modelling (ENM). Ecological niche models use data of known occurrences of a species (realized niche, RN) and its current environmental envelope to determine what environmental conditions are significant proxies for constraint of species' range [28,29]. The current environmental envelope of a species is estimated using environmental covariate data in a correlative statistical modelling framework. The model is then used to hindcast or forecast the potential distribution of the species of interest (potential niche, PN), providing an estimate of where these environmental conditions are most likely to occur in the past or future. Inferences from niche modelling will be limited if sampling of presence data is non-random and absence data is lacking [29], and is only useful for predicting future distributions if the environmental variables included are relevant and life-history characteristics are taken into account [28]. To improve model predictability, ensemble modelling is commonly used in ENM. Multiple statistical models are applied to datasets, and predictive outputs meeting an evaluation threshold are then combined in a single ensemble model [30]. Ensemble modelling allows the user to capture predictive signal from multiple models, reducing over-reliance on the assumptions of individual algorithms [30,31].

We examined environmental constraints on an endemic radiation of grasshoppers in *Aotearoa* New Zealand that belong to the acridid family Catantopinae [32]. Before the arrival of humans about 800 years ago [33], most of the New Zealand landscape was clothed in forest while open habitat was limited to high-elevation or semi-arid hinterland and braided riverbeds [34]. Though relatively scarce, these open areas support a rich biota with high species endemism [35]. The alpine zone in particular exerts strong selective pressure on the fauna and flora resulting in specialized adaptations. For example, many New Zealand alpine insects are freeze-tolerant, including the grasshoppers in this study, which can survive equilibrium freezing year round and at any life-history stage [36]. Grasshoppers are suitable representatives of the alpine fauna, because their distributions are closely associated with climatic gradients that predict the availability of open habitat [37,38], they display alpine adaptations and have limited dispersal capacity due to a lack of functional wings. They are therefore particularly vulnerable to the types of environmental change exerted by climate warming that is being documented around the globe [39]. We model the environmental envelope of 12 species of New Zealand flightless grasshoppers (10 alpine species and two lowland relatives) using presence and absence data in conjunction with environmental covariate data in a statistical modelling framework. We assess the potential impacts of climate change on projected distributions by transferring the ensemble models to future climate change scenarios to predict how their optimal habitat might grow, shrink and/or fragment over the next 50 years of climate change.

## 2. Material and methods

### 2.1. Collection of species location records

**Figure 1.**
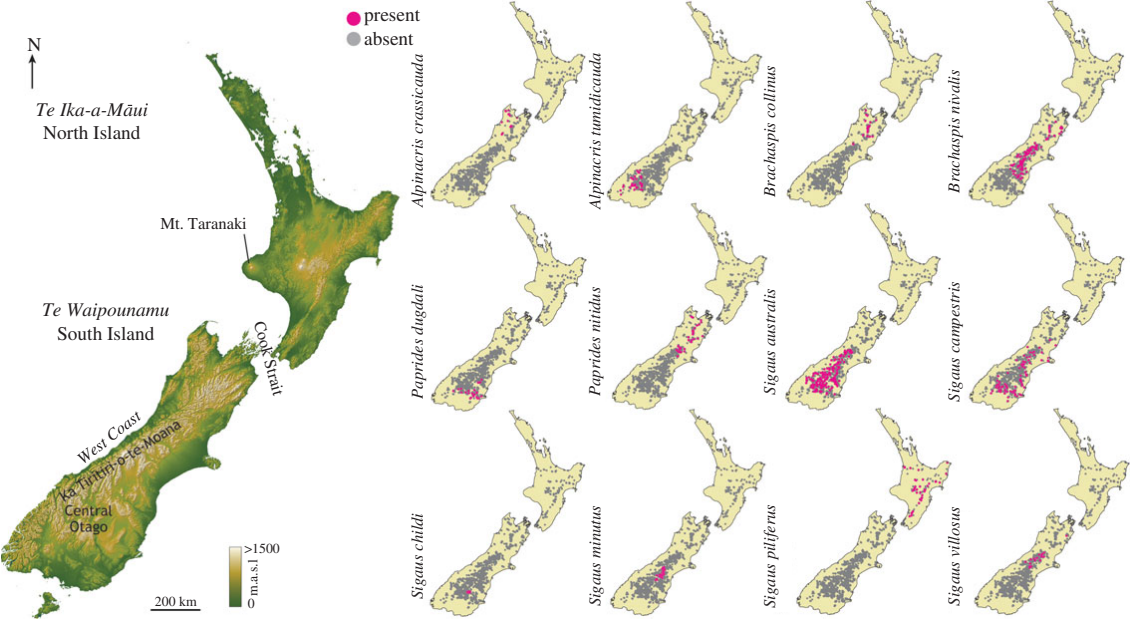
Relief map of *Aotearoa* New Zealand with locations referred to in the text (left). Distribution maps (right) show the locations of presence and absence data points input into ecological niche models for each of 12 New Zealand grasshopper species (Acrididae; Catantopinae).

For 12 grasshopper species, location records were collated from insect collections, journal articles, theses, books and Crown Pastoral Lease Tenure Reviews (CPLTR) (electronic supplementary material S1) published between 1967 and 2016. These 12 endemic New Zealand grasshoppers consist of 10 high-elevation species: *Alpinacris crassicauda*, *Alpinacris tumidicauda*, *Brachaspis collinus*, *Brachaspis nivalis*, *Paprides dugdali, Paprides nitidus*, *Sigaus australis*, *Sigaus campestris*, *Sigaus piliferus* and *Sigaus villosus*, and two low-elevation relatives: *Sigaus childi* and *Sigaus minutus*. Given the range of sources used to obtain location data, our sample does not suffer from the common bias of only collecting from sites close to access roads. In particular, our location data benefit from the use of CPLTR that are produced by Land Information New Zealand and contain conservation reports and ecological surveys carried out by the Department of Conservation on pastures throughout *Te Waipounamu* (South Island), New Zealand. Additional records were retrieved from our specimen collections at Massey University. World Geodetic System 1984 (WGS84) coordinates (latitudes and longitudes) were obtained for each location record using NZ Topo Map [40]. Location records for two additional endemic lowland grasshopper species (*Phaulacridium marginale* and *Phaulacridium otagoense*) were included in the dataset, but not modelled in this study [37]. Both of these species have low-elevation distributions and provided additional location searches and absence data that increase the accuracy of the ENMs. Coordinates were reduced to two decimal places and duplicate records within species were removed, in order to ensure only one record per approximately 1 km^2^ for each species. Coordinates were also mapped for each species to ensure there were no outliers due to incorrect species identification. Following filtering, a total of 949 location records were available for our analysis (figure 1). All location records and their sources can be found in electronic supplementary material S1.

### 2.2. Predictor variable layers

In order to define and project the PN of each grasshopper species, 19 bioclimatic variables were obtained from the Worldclim v. 1.4 database for two time periods—current (e.g. data averaged from 1960 to 1990) and future (e.g. data predicted and averaged from 2061 to 2080; electronic supplementary material, S2), at a resolution of 30 arc-seconds (approximately 1 km^2^) [41–43]. Two versions of predicted climate variables for the future were acquired, derived from representative concentration pathway (RCP) 2.6 and RCP8.5 models [44]. These represent potential low and high greenhouse gas concentration scenarios, respectively (electronic supplementary material, S3), and mean global temperature increases of 1.0°C (RCP2.6) and 3.7°C (RCP8.5). All climate layers were generated using the MIROC-ESM global climate model [45]. The bioclimatic layers were cropped in QGIS v. 2.16.1 [46] to the extent of New Zealand between latitudes −49° and −32° and longitudes 165° and 180°. To reduce variable collinearity and minimize model over-fitting, a variance inflation factor analysis was implemented in R using the ‘usdm’ package, applying a threshold of 0.85 [47]. This resulted in retention of seven independent ecologically informative bioclimatic variables.

Two additional environmental data layers were acquired from the New Zealand Land Resource Information System portal (https://lris.scinfo.org.nz/): fundamental soil layers New Zealand Soil Classification and Vegetative Cover Map of New Zealand [48,49]. The soil and vegetation data layers were rasterized, clipped and rescaled from the original files. These variables have previously been found to contribute significantly to models of grasshopper distribution [50,51]. These layers represent the current approximate state of vegetation and soil in New Zealand, and, as no future (e.g. 2070) GIS files exist for soil and vegetation cover in New Zealand, they were used as static layers throughout the modelling process. Ecological niche models that include such static layers are known to perform as well as, or better than, models where only dynamic variables are included [52].

### 2.3. Ecological niche models

We used ENM analyses implemented in the R package ‘biomod2’ v. 3.3–7 [53]. Ten different statistical modelling methods were applied to the nine environmental variables and presence/absence data of each individual grasshopper species: generalized linear model (GLM), generalized boosting model/boosted regression tree (GBM), generalized additive model (GAM), classification tree analysis (CTA), artificial neural network (ANN), surface range envelope (SRE), flexible discriminant analysis (FDA), multiple adaptive regression splines (MARS), random forest (RF) and maximum entropy (MAXENT). The Biomod2 BIOMOD_Modeling() function was used to initially run each statistical model, with all model parameters kept at default values. Eighty per cent of the input data were used to calibrate the models, with the remaining 20% used to test them. The Prevalence parameter was set at 0.5, allowing absences and presences to be weighted equally and VarImport was set to 3, allowing three permutations to estimate variable importance. Calibration and evaluation of each statistical model was repeated three times, resulting in 30 models per species. A summary of raw, clean, training and testing absence and presence points input into the models can be found in the electronic supplementary material, S4.

Model evaluation of the 30 ENMs for each species was carried out using two methods: receiver operating characteristic (ROC) and true skill statistic (TSS). Models with ROC values of 0.9–1 and TSS values of 0.8–1 are considered to have ‘excellent’ predictive power (accuracy) [54]. Following initial modelling and model evaluation, the Biomod2 BIOMOD_EnsembleModeling() function was used to formulate ensemble models for each species. Ensemble models were built using the ‘all’ total consensus model option, with the eval.metric set to ROC, and eval.metric.quality.threshold set to 0.9, so only models with ROC scores greater than 0.9 (or greater than 0.8 for three species) were used in building the ensembles. The BIOMOD_Projection() function was then applied, projecting ensemble forecasts for the two future climate scenarios. Plots were subsequently made for the three datasets (current, RCP2.6 and RCP8.5) using the ensemble model weighted mean model (EMwm) output. EMwm variable importance was calculated by applying the weights produced in the ensemble model to the variable importance results of the initial 30 model dataset. These were then averaged across the three model runs, and EMwm variable importance was calculated by summing the total of these averages for each predictor variable and dividing by the number of modelling methods used (10) [55]. Final scores of variable importance were converted into percentages, with higher percentages indicating a variable was more influential in enabling models to estimate the niche of a particular species [56].

### 2.4. Fragmentation

Binary raster layers of each ENM were generated for range change and fragmentation statistical analyses. These binary vectors were generated from the 30 niche model dataset, where each pixel that scored greater than a predetermined cut-off value in greater than 50% of models was ranked as a 1, and all other pixels as 0. When comparing binary vectors between current and future models, Biomod2 ranks pixels as either, ‘Never occupied’ (pixels were unoccupied and remain unoccupied between models), ‘Always occupied’ (pixels were occupied and remain occupied between models), ‘Lost’ (pixels were occupied but become unoccupied in the future RCP scenario) or ‘Gained’ (pixels that were unoccupied in the current model become occupied in the RCP model). From this information range change statistics were calculated with two contrasting models. The dispersal model assumed the species in question will occupy any pixels ‘gained’ in future models, implying no limitation on dispersal and/or establishment; the model essentially treats potential habitat as realized habitat. At the other extreme, the non-dispersal model precluded occupation of disconnected ‘gained’ pixels, which means nearby but separate habitat patches as well as more distant patches (e.g. on a separate island) are assumed to be uncolonized. We also applied FRAGSTATS, implemented in R package ‘SDM Tools’ v. 1.1-221 [57] (see McGarigal [58] for a detailed description of FRAGSTATS metrics), to the binary files to estimate fragmentation statistics that can be compared between different RCP scenarios from their current niche models. Pixels that are connected within each of the binary files are given unique patch identities, and the area and number of these patches calculated for each scenario. We excluded small patch fragments that were less than 0.1 km^2^ from the fragmentation statistics analysis. Methods and scripts implemented in this study can be found in the electronic supplementary material S5 and S6.

## 3. Results

After filtering, nine of the 12 grasshopper species had more than 30 location records as ‘present’ (table 1). A minimum of 30 occurrences are recommended for ENMs, below which model accuracy decreases, and variance of model accuracy increases [59–62]. The majority of evaluation scores averaged across the three model runs were greater than 0.9 ROC (65/120), but for the four most well-sampled and widespread species (*B. nivalis*, *P. nitidus*, *S. australis* and *S. campestris*), no individual model had a ROC score greater than 0.9 (electronic supplementary material, S7). Fewer models had TSS scores greater than 0.8 (55/120), with none gaining a TSS greater than 0.8 for *B. nivalis*, *S. australis* and *S. campestris*. The models that most commonly had the highest model evaluation scores across species were GBM, followed by MAXENT and RF, while CTA, ANN and SRE models consistently had low evaluation scores across species (electronic supplementary material, S7). For the species with ROC scores below the 0.9 threshold, we applied a ROC threshold of greater than 0.8 to formulate EMwm ensemble models. For most of the grasshopper species, the EMwm ensemble model had an improved ROC score compared with individual model outputs (electronic supplementary material, S7). The exception was the rare and localized grasshopper *S. childi*, whose RF model and EMwm model could not be improved upon (both had a ROC score of 1). The EMwm models for *B. nivalis*, *S. australis* and *S. campestris* had ROC scores greater than 0.9, improving on the predictive accuracy of their individual models. Ten species (*A. crassicauda*, *A. tumidicauda*, *B. collinus, B. nivalis, P. dugdali*, *P. nitidus, S. childi*, *S. minutus*, *S. piliferus* and *S. villosus*) had EMwm sensitivity scores (percentage correctly predicted presences) greater than 90%. Seven of these species also had specificity scores (proportion of correctly predicted absences) greater than 90% (electronic supplementary material, S7). The two species, *S. australis* and *S. campestris*, for which niche models had the poorest fit (i.e. scored less than 90% for both sensitivity and specificity) were both well-sampled, widespread, grasshoppers. In the case of *S. campestris*, this relatively low model fit can be explained by the availability of suitable habitat in North Island which is out of reach of this flightless insect (the assumption that realized distribution encompasses most of potential habitat is therefore invalid in this case). For *S. australis*, it is possible that the assumption of niche stability and conservatism has been invalidated by recent range shifts associated with anthropogenic land-use change [38].

**Table 1.** Environmental characteristics of 12 flightless, alpine New Zealand grasshopper species modelled in this study. The most important predictor of species distribution (from nine variables) is shown for each species (EMwms). Predictor variable importance scores for all models and all species are in electronic supplementary material, S8. Range-change percentages are based on the loss/gain/stability of pixels between vector binary maps (electronic supplementary material, S9). Predicted range changes include all gained pixels (with dispersal), while range change with no dispersal includes only gain of currently occupied habitat fragments.

species	elevation range (m.a.s.l)	number of presence points	number of absence points	most important predictor variable	% range change from present (with dispersal)		% range change from present (no dispersal)	
					RCP2.6	RCP8.5	RCP2.6	RCP8.5
*A. crassicauda*	975–1680	16	933	mean diurnal range	−43	208	−61	−11
*A. tumidicauda*	600–1830	49	900	precipitation of driest month	−74	−35	−74	−37
*B. collinus*	1000–2000	30	919	mean diurnal range	−95	−66	−96	−89
*B. nivalis*	450–2000	93	856	isothermality	59	134	−9	−7
*P. dugdali*	400–1160	29	920	precipitation seasonality	−75	−96	−82	−97
*P. nitidus*	600–1830	91	858	mean temp. of driest quarter	12	17	−61	−35
*S. australis*	285–2020	278	671	annual mean temp.	−74	−93	−75	−93
*S. campestris*	0–1550	100	849	soil	−45	−67	−45	−67
*S. childi*	160–420	41	908	precipitation of driest month	−47	154	−100	−89
*S. minutus*	500–1180	40	909	mean diurnal range	4	191	−80	−44
*S. piliferus*	725–1100	45	901	mean temp. of wettest quarter	−15	−58	−45	−77
*S. villosus*	1370–2130	23	926	annual mean temp.	168	−77	−20	−94

**Figure 2.**
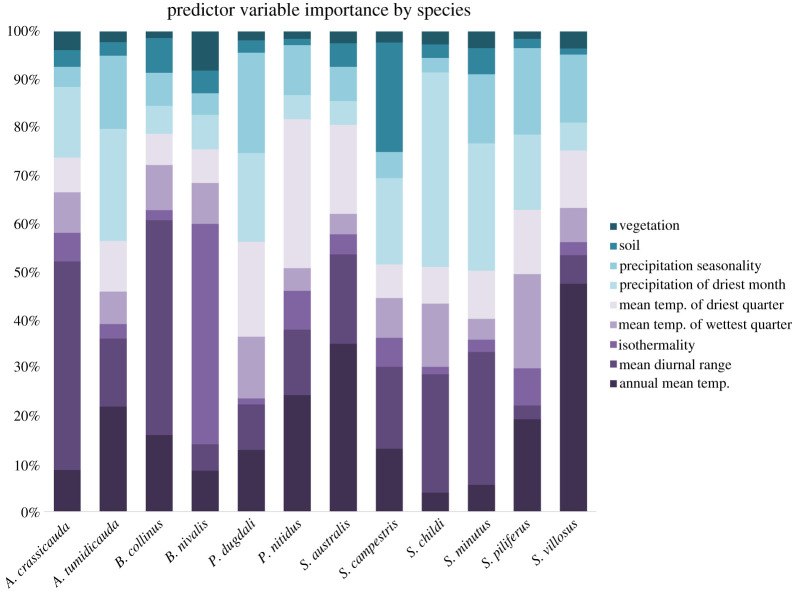
Environmental predictor variables differ in their importance in models of presence for 12 species of New Zealand alpine grasshoppers in the genera *Alpinacris*, *Brachaspis*, *Paprides* and *Sigaus*. Relative scores (percentages) are from the EMwm. See electronic supplementary material, S8 for results of other models.

Considering the EMwm models only, eight of the nine predictor variables were important in explaining the distributions of the 12 grasshopper species (see electronic supplementary material, S8 for other models). Vegetation did not rank as an important predictor variable for any species and soil was influential for only one species, *S. campestris* (table 1 and figure 2). Temperature was an important predictor of habitat for most species (annual mean temperature, mean diurnal range and mean temperature of driest quarter). Water availability given as precipitation of driest month and precipitation seasonality were influential for three species (table 1). For one species, isothermality was the most important predictor (*B. nivalis*). Despite passing the plausibility criteria, it must be remembered that all of these variables are likely to be proxies for a combination of environmental traits.

### 3.1. Models for current conditions

**Figure 3.**
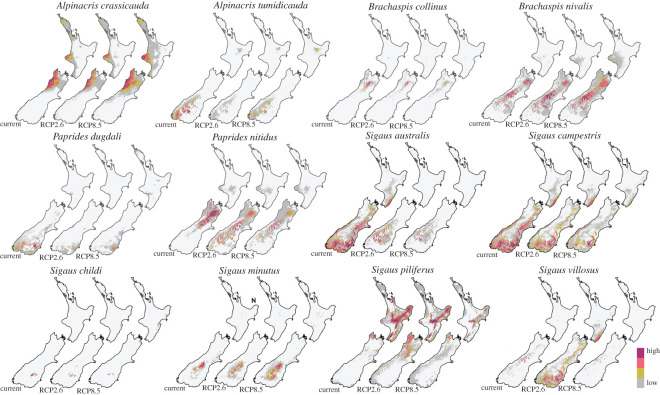
The realized and predicted niche space of 12 New Zealand alpine grasshopper species generated by ENM (EMwms). Maps show ecological niche models for current climate and as predicted for two future climate change scenarios (RCP2.6 (1°C rise) and RCP8.5(3.7°C rise) trajectories). Colours indicate probability (high to low) of suitable habitat under the model.

Initial modelling using presence/absence records and current environmental conditions revealed where suitable habitat currently exists for each species (RN). This was compared with the known distribution of each species in order to determine if the models are ecologically plausible (figure 3). The modelled potential distribution of eight species (*A. tumidicauda*, *B. collinus*, *B. nivalis, P. dugdali*, *P. nitidus*, *S. australis*, *S. childi, S. minutus* and *S. villosus*) closely matched their current occupancy, indicating that their current distribution is probably constrained by the climate variables or their proxies in the models. For three species (*A. crassicauda*, *S. campestris* and *S. piliferus*), modelled potential distribution was larger than their current distributions. Of these three, all species had projected habitat that expanded onto the main island (North or South) where they are currently not found. Additionally, the projected suitable habitat of *S. campestris* and *S. piliferus* was found to be larger than their current distribution within their respective islands. Our model predicts suitable climatic environment to exist further west and north than these species have been recorded. Suitable climate for *S. piliferus* is suggested in the west (Mt Taranaki), and also along the southern east coast *Te Ika-a-Māui* (North Island), where the species has not been recorded.

### 3.2. Future ecological niche models

**Figure 4.**
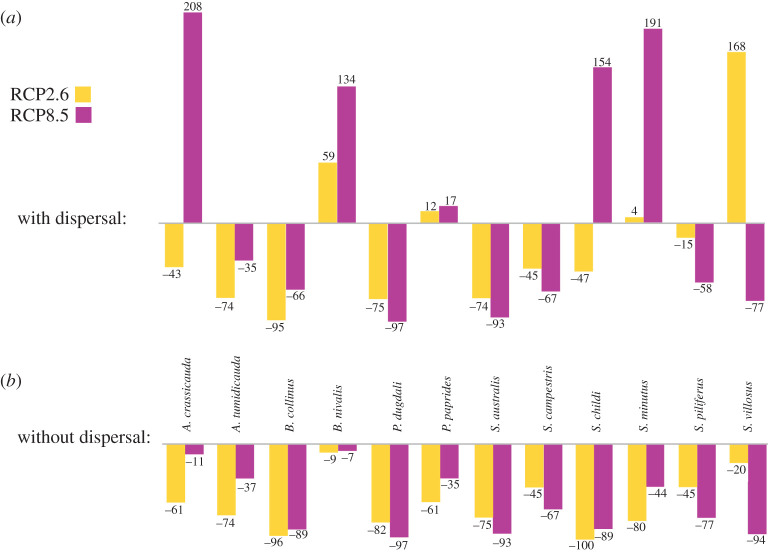
Predicted loss of suitable habitat (as a percentage of current range) for 12 grasshopper species in the New Zealand alpine genera *Alpinacris*, *Brachaspis*, *Paprides* and *Sigaus* under two future climate scenarios: approximately 1.0°C rise by 2070(RCP2.6) and approximately 3.7°C rise by 2070(RCP8.5). Percentages are based on the loss/stability of pixels between vector binary maps (current models compared with future); (*a*) is the predicted loss/gain of all potential habitat patches and (*b*) is the predicted loss of space excluding fragments that would require dispersal by these flightless grasshoppers to newly available pixels (habitat).

Changes in the size of each grasshopper projected niche (PN) were calculated for both RCP scenarios (electronic supplementary material, S9). Some species were predicted to lose habitat while other species were predicted to gain PN (table 1 and figure 4; electronic supplementary material, S9). With a mean temperature increase of 1°C by approximately 2070 (RCP2.6), eight species (*A. crassicauda*, *A. tumidicauda*, *B. collinus*, *P. dugdali*, *S. australis*, *S. campestris*, *S. childi* and *S. piliferus*) are predicted to lose PN, while four species are predicted to gain PN *(B. nivalis, P. nitidus*, *S. minutus* and *S. villosus*). Of those that will lose PN, four species (*A. tumidicauda*, *B. collinus, P. dugdali* and *S. australis*) will lose greater than 50%, with the greatest loss being 95% of PN for *B. collinus*. *Sigaus villosus*, however, is predicted to gain 168% of PN, while *B. nivalis, P. nitidus* and *S. minutus* gain 59%, 12% and 4% in our models, respectively. However, with a mean temperature increase of 3.7°C by approximately 2070 (RCP8.5), seven species (*A. tumidicauda*, *B. collinus*, *P. dugdali, S. australis*, *S. campestris, S. piliferus* and *S. villosus*) are predicted to lose PN, with six species (*B. collinus*, *P. dugdali*, *S. australis*, *S. campestris*, *S. piliferus* and *S. villosus*) predicted to lose over 50%, and one species (*A. tumidicauda*) predicted to lose 35% of its predicted PN. Five species (*A. crassicauda*, *B. nivalis*, *P. nitidus*, *S. childi* and *S. minutus*) gain PN in our models, with three species (*A. tumidicauda*, *S. childi* and *S. minutus*) gaining over 150%.

As all grasshopper species in this study are flightless, dispersal between habitat patches will be limited. When niche space was recalculated to exclude newly available (gained) niche space that would require dispersal between fragments, all species were predicted to lose PN in the future (table 1). With a mean temperature increase of 1°C by approximately 2070 (RCP2.6), eight species (*A. crassicauda*, *A. tumidicauda*, *B. collinus*, *P. dugdali*, *P. nitidus*, *S. australis*, *S. childi* and *S. minutus*) will lose greater than 50% of PN if they are unable to disperse. Three other species (*S. campestris, S. piliferus* and *S. villosus*) will lose over 20% of their predicted PN. The greatest loss will be by *S. childi* (−100%), followed closely by *B. collinus* (−96%). With a mean temperature increase of 3.7°C by approximately 2070 (RCP8.5), seven species (*B. collinus*, *P. dugdali*, *S. australis*, *S. campestris*, *S. childi, S. piliferus* and *S. villosus*) will lose over 50% of their predicted PN, and three species (*A. tumidicauda*, *P. nitidus* and *S. minutus*) will lose over 35%. *Paprides nitidus* is predicted to have the greatest loss in this scenario (−97%), followed closely by *S. villosus* (−94%) and *S. australis* (−93%).

### 3.3. Fragmentation statistics

**Figure 5.**
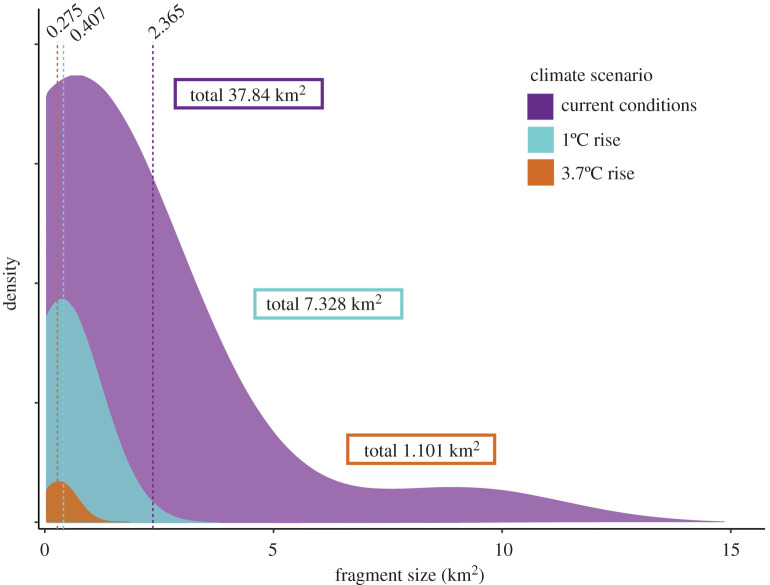
Predicted habitat fragmentation for the endemic, flightless, alpine grasshopper *Sigaus australis* on *Ka Tiritiri-o-te-Moana*, *Aotearoa* (Southern Alps, New Zealand). Density distributions of habitat patches greater than 0.1 km^2^ in current conditions and two future scenarios under anthropogenic climate change (RCP2.6 and RCP8.5). Vertical dashed lines indicate means of each distribution with their values. Density plots are scaled to sum total of habitat greater than 0.1 km^2^ under each scenario (see electronic supplementary material, S12 for plots of other species).

Fragmentation statistics were generated for current and the two future RCP scenarios based on vector binary maps (figure 5; electronic supplementary material, S10.1; S10.2; S11; S11; S12). Patches of connected ‘suitable habitat’ pixels were identified in each map and given unique IDs from which FRAGSTATS were calculated. The most common trend between current and future habitat fragmentation statistics was a decrease in the mean patch size associated with a decrease in suitable total habitat area. In some instances (11/24) of the scenarios across species: *A. crassicauda* (RCP2.6), *A. tumidicauda* (RCP2.6), *B. collinus* (RCP2.6 and RCP8.5), *P. dugdali* (RCP2.6 and RCP8.5), *S. australis* (RCP8.5), *S. campestris* (RCP2.6 and RCP8.5), *S. piliferus* (RCP8.5) and *S. villosus* (RCP8.5), a decrease in patch number was evident in an increase in the splitting index and/or decrease in the aggregation index in future scenarios (c.f. current conditions), indicating an increase in fragmentation and an increase in distance between patches, respectively. In three scenarios (*A. tumidicauda* (RCP8.5), *S. australis* (RCP2.6) and *S. piliferus* (RCP2.6)) where there was a decrease in the total area of patches, there was a decrease in mean patch size and increase in the number of patches. And for another three scenarios (*P. nitidus* (RCP2.6 and RCP8.5) and *S. childi* (RCP8.5)) where there was an increase in total patch area, there was a decrease in the mean area of patches, but an increase in the number of patches.

## 4. Discussion

Patterns of biodiversity are intrinsically linked to changes in Earth's climate [63,64]. Long-term climate change is expected to result in species becoming more isolated, experiencing phenological mismatch, losing suitable habitat and encountering new competition and community assemblages as ranges shift [5]. Whether a species survives such changes or is extinguished depends on the severity and speed of change in climate, and the adaptive capacity of the species involved [65]. The pace of anthropogenic climate change [39] imposes intense selective pressure on many taxa, and those on steep climatic gradients are likely to be influenced most rapidly [4–6]. This is the situation for alpine grasshoppers endemic to *Aotearoa* New Zealand. Comparison of their current distributions with modelled suitable niche space reveals that climate is a primary factor controlling their distribution. Despite the ecological overlap of New Zealand's alpine grasshopper species, the environmental proxies/variables that best explain their current distributions were species specific. It is likely that changes in species distributions resulting from global warming will also be species specific, but most New Zealand grasshoppers will be negatively affected. Furthermore, for most species, we identified a decrease in the total area of suitable habitat patches and reduction in the mean size of these patches and the total number of patches, which will result in population fragmentation and vulnerability to stochastic loss and erosion of intra-specific diversity [66,67]. Despite New Zealand's alpine habitat being less than 10 million years old, it is home to an extensive array of specialized, endemic plants and insects [14,35], all of which will be vulnerable to climate change. Given our finding of idiosyncratic responses of individual grasshopper species, we recommend ENM be applied to other alpine specialists such as crickets *Pharmacus* spp. (Rhaphidophoridae) and penwiper plants *Notothlaspi* spp. (Brassicaceae) in order to identify whether species extinctions could be prevented with human-mediated dispersal of population samples to unoccupied alpine habitat.

### 4.1. Ecological niche modelling

ENM will only work well when species are limited by abiotic environmental factors rather than by species interactions and their dispersal ability to access suitable conditions. In cases where RN is close to fundamental niche, we can expect high performance of models if distribution data are adequately sampled and all relevant environmental drivers are included in the models. The predictive accuracy of ecological niche models is dependent on the number and distribution of presence records available for each species and the quality of absence information [59,60,62]. Here, each grasshopper species was represented by presence data that closely reflected the spatial ranges of each species, and the accumulation of observations of 14 New Zealand grasshopper species provided the advantages of accurate absence data. For some species (*P. dugdali*, *S. piliferus* and *S. villosus*), the potential for additional data points is limited because they have restricted and patchy occurrence in current conditions. In fact, it was those species with the highest numbers of presence data points (e.g. *B. nivalis*, *P. nitidus, S. australis* and *S. campestris*) that yielded models with the lowest sensitivity and specificity scores. Although seemingly contrariwise, the tendency for the distributions of rare or spatially restricted species to yield statistically more robust models than common or widely distributed species [56,68,69], reflects the detection of more environmental variance across wide-ranging locations. This might result from the inadvertent pooling of distinct lineages (cryptic taxa) adapted to different conditions, and with the potential to respond in different ways to environmental change. In such instances it may be beneficial to model phylogenetic lineages separately to improve the predictive accuracy of models [70–72].

With the available data, the individual statistical models generated were generally well supported by both ROC and TSS model evaluation metrics. Across species, a minimum of four statistical models that met evaluation thresholds were incorporated into ensemble models, although the combination of models varied by species. Evaluation metrics for the final ensemble models indicate the ensembles out-performed individual models (the exception being *S. childi* where they were equal), and this is consistent with other studies [31,59,73]. This emphasizes the importance and benefits of exploring a range of statistical models and applying the ensemble model approach. We did not need to rely on a single algorithm to model our predictions and different ensembles of statistical models could be applied depending on what was most relevant for each species.

Of the climate, vegetation and soil inputs applied in this study, climate variables were predominantly the most important at explaining the distribution of the grasshopper species. For most species, a single predictor variable tended to be a more important environmental proxy than any other. In the majority of instances, the current distribution of grasshopper species matched the ecological niche space predicted by the ENMs. A mismatch between the current distribution of a species and its predicted niche under current conditions indicates that other variables are limiting the distribution of these species [74]. This could involve abiotic variables not included in the analysis, or biological interactions such as competition, predation, disease and/or parasitism. Biotic variables are notoriously difficult to account for in ENM [74]. Climate is evidently a key driver in the distribution of New Zealand's endemic alpine grasshoppers. When transferring ecological niche models to future climate scenarios we assumed that environmental correlations remained the same as those determined for current New Zealand climate variables, but additional aspects including changes in anthropogenic land use will need consideration for protection of biodiversity.

### 4.2. Future climate

Predictions of how species may fare with future climate change varied, depending on the RCP scenario, and whether or not ability to disperse across habitat gaps was taken into account. Most species were predicted to lose at least 30% of suitable habitat under both RCP scenarios (table 1; electronic supplementary material, S8). In the last 20 years, global mean surface temperature has already increased by 0.66°C [75], suggesting that a rise of 1°C (RCP2.6) is an underestimate of the speed and extent of warming Earth's biota will experience. Even under this conservative scenario, the currently widespread alpine species *B. collinus* will lose 95% of its range, putting it at high risk of extinction. Other grasshopper species were predicted to gain PN from a rise of 1°C (RCP2.6), while also being predicted to lose PN from a rise of 3.7°C (RCP8.5), and vice versa. Furthermore, closely related species did not demonstrate similar responses to change. An example is the endangered species *S. childi* which currently has a narrow distribution in Central Otago of the South Island within the much wider range of its sister species *S. australis*. *Sigaus childi* is specialized to low-elevation semi-arid environments in Central Otago, while *S. australis* is primarily an alpine species with a range now extending to low elevations. With a rise of 3.7°C (RCP8.5), *S. childi* was predicted to gain substantial niche space, as semi-arid conditions expand in the Central Otago region, although only after losing 47% of its habitat at +1°C. By contrast, *S. australis* was predicted to lose projected niche space under all scenarios. Perhaps *S. childi* has the potential to expand its range and out-compete *S. australis* in the future (although hybridization might prevent this [76]). Of more immediate concern for this endangered species is that without the ability to colonize new habitat patches, *S. childi* is predicted to go extinct unless we invest in human-mediated dispersal. When no-dispersal was applied to the range-change calculations, no New Zealand grasshopper species maintained its range, with all species losing PN. Full dispersal and no dispersal are the extreme assumptions to apply to these models [77], and it is likely that even with their poor dispersal abilities, the grasshoppers have some potential to move short distances to colonize new patches. This would not, however, allow shifts between habitat on different mountain ranges or islands of *Aotearoa*, and the general pattern seen across grasshopper species in this study, was of negative range outcomes with climate change, as found for alpine invertebrates around the world [78–80].

The extent and consequences of PN loss will be species specific. The impact of a reduction of 10% of PN would probably be negligible for species that currently have wide distributions (e.g. *S. australis*, *P. nitidus*), compared with range-restricted species (e.g. *S. childi*). Despite being predicted to gain PN under various scenarios, species with lowland distributions (*S. childi* and *S. minutus*), will be at greatest risk of land-use change including conversion of native grasslands for agricultural land, invasion of exotic plants (e.g. *Radiata* pine) and fire, with the end result likely to be just as detrimental as for high-elevation species [37].

### 4.3. Fragmentation results and consequences

Habitat fragmentation typically results in reduced net population size and changes in metapopulation dynamics (e.g. [81]) that can result in a loss of genetic diversity through genetic drift and inbreeding depression [82–84]. By contrast, population fragmentation is also hypothesized as a mechanism generating biodiversity hotspots associated with small-island effects, including in alpine areas [85,86]. The outcomes of habitat fragmentation for inter- and intra-specific diversity are sensitive to many factors [87–89], but rapid, sustained fragmentation resulting in patch size reduction when coupled with limited dispersal ability will negatively affect the ability of species to track optimal conditions under future climatic change pressure.

As New Zealand's endemic alpine grasshoppers are flightless, and New Zealand is divided into two main islands, it is important to consider that even though new PN space might become available in the future, the grasshoppers may not be able to disperse into these new areas without human assistance. Evidence of this can be seen in models of current suitable niche space. For example, there is predicted to be suitable habitat for *A. crassicauda* and *S. campestris* in the North Island, but these species remain only distributed in the South Island. For *S. piliferus*, suitable habitat is available in the South Island, but this species is limited to the North Island. During the last glacial maximum (LGM), the North and South Islands were connected by lowland habitat across the Cook Strait—however, even this did not facilitate the movement of South Island species northwards or vice versa (or they have subsequently been extirpated) [90]. Even within a land area grasshopper ranges are restricted to suitable habitat, for example *S. piliferus* is predicted to exist on Mt Taranaki on the west of the North Island; however, this species has never been recorded there. The inability of these endemic New Zealand grasshoppers to readily disperse (i.e. via flight) is already a limiting factor in their current distributions.

The fragmentation statistics estimated from the ENMs predict that habitat for the grasshoppers will be increasingly fragmented in the future—regardless of predicted total habitat area. In most cases, future total area of patches was predicted to decrease, with patches either reducing in number as well as size, or reducing in size but increasing in number. Even when overall area was predicted to increase, this was accompanied by an increase in the number of patches (i.e. increased fragmentation) (figure 5). New Zealand's alpine environments have been more connected in the past—most recently during the LGM ending approximately 15 kya [38], with the ranges of many species subsequently receding and fragmenting. Around the world, many alpine ecosystems are recognized hotspots of endemism, hypothesized to be as a result of steady climate cycling, habitat fragmentation and species diversification [85,86]. Biodiversity outcomes from environmental fluctuation and species adaptation over tens or hundreds of thousands of years are not comparable with change occurring within 100 years. The rate of habitat loss and fragmentation predicted by 2070 in this study may be too fast for many alpine species to adapt. The alpine grasshoppers analysed here have approximately 50 generations to shift ranges, niches or adapt *in situ* to predicted changes. Furthermore, if humans are unable to curb carbon emissions, the consequences may be worse than what we have predicted here [39].

## 5. Conclusion

By modelling the environmental envelopes of New Zealand's endemic alpine grasshopper fauna, and the likely changes to local environmental conditions under future climate change scenarios, we witness the complexities in predicting the distributions of species in the future, despite shared ecosystems and history. We have determined that the overall trend for this group will be a decrease in projected niche space by 2070; however, there are some exceptions, and sensitivity of models to different warming scenarios varies. We demonstrate the importance of considering the dispersal ability of species when inferring predictions, in addition to fragmentation and total area of PN, as this could greatly impact the chances of species surviving future climate change and land-use changes.

## Data Availability

Specimen locality data are presented in the electronic supplementary file. Environmental input and coding information are available in file Biomod2_Archive at the website: evolves.massey.ac.nz. Link: http://evolves.massey.ac.nz/Data/Biomod2_Archive.zip. The data are provided in the electronic supplementary material [91].
